# Quantitative DWI predicts event-free survival in children with neuroblastic tumours: preliminary findings from a retrospective cohort study

**DOI:** 10.1186/s41747-019-0087-4

**Published:** 2019-01-30

**Authors:** Anna-Lydia Peschmann, Meinrad Beer, Bettina Ammann, Jens Dreyhaupt, Katharina Kneer, Ambros J. Beer, Christian Beltinger, Daniel Steinbach, Holger Cario, Henning Neubauer

**Affiliations:** 1 Department of Diagnostic and Interventional Radiology, University Hospital Ulm, Albert-Schweitzer-Allee 23, 89081 Ulm, Germany; 2 Department of Biometrics, University Hospital Ulm, 89081 Ulm, Germany; 3 Department of Nuclear Medicine, University Hospital Ulm, 89081 Ulm, Germany; 4 Department of Paediatrics and Adolescent Medicine, University Hospital Ulm, 89081 Ulm, Germany

**Keywords:** Apparent diffusion coefficient, Diffusion magnetic resonance imaging, Ganglioneuroblastoma, Ganglioneuroma, Neuroblastoma

## Abstract

**Background:**

Quantitative diffusion-weighted imaging (DWI) probes into tissue microstructure in solid tumours. In this retrospective ethically approved study, we investigated DWI as a potential non-invasive predictor of tumour dignity and prognosis in paediatric patients with neuroblastic tumours.

**Methods:**

Nineteen consecutive patients with neuroblastoma (NB, *n* = 15), ganglioneuroblastoma (GNB, *n* = 1) and ganglioneuroma (GN, *n* = 3) underwent 3-T magnetic resonance imaging at first diagnosis and after 3-month follow-up, following a protocol including DWI (*b* = 50 and 800 s/mm^2^) in addition to standard sequences. All DWI scans were analysed for tumour volume assessment and apparent diffusion coefficient (ADC) calculation. Correlation with tumour pathology and risk factors (bone-marrow metastases, MYCN-amplification and 1p-deletion), therapeutic regime (observation *versus* chemotherapy) and clinical follow-up was evaluated.

**Results:**

At baseline, mean ADC in NB was lower than in GNB/GN (0.76 vs. 1.47 × 10^−3^ mm^2^/s, *p* = 0.003). An ADC cutoff ≤ 1.05 identified malignant disease with 100.0% sensitivity (95% confidence interval [CI] 29.2–100.0%) and 93.8% specificity (95% CI 69.8–99.8%). Initial ADC was < 0.80 in all NB patients with eventual tumour relapse. During follow-up, tumour ADC values increased in the observation group (NB/GN) without relapse (*p* = 0.043). In eventually relapsing tumours, ADC values at follow-up tended to decrease further despite reduction in tumour volume.

**Conclusions:**

ADC values at first presentation differed significantly between malignant and benign neuroblastic tumours. Low baseline ADC was predictive of tumour progression and relapse in NB patients. With therapy, increasing ADC values appeared to predict relapse-free survival, while a decreasing ADC during therapy was an indicator of poor prognosis.

## Key points

- Diffusion-weighted imaging (DWI) at 3 T provides a promising biomarker in paediatric neuroblastic tumours
- Baseline apparent diffusion coefficient (ADC) values are helpful for non-invasive prediction of tumour dignity
- A high ADC is predictive of event-free survival
- Tumour risk factors may be mirrored by lower ADC values

## Background

Neuroblastic tumours arise from cells of the peripheral sympathetic nervous system and are classified as ganglioneuroma (GN), ganglioneuroblastoma (GNB) or neuroblastoma (NB) [1]. Neuroblastoma (NB) constitutes the most common extracranial solid malignancy in children with highly variable biological potential ranging from spontaneous tumour regression to highly aggressive growth and metastatic spread [2–4]. While magnetic resonance imaging (MRI) is the preferred cross-sectional imaging modality for diagnostic work-up of NB patients, standard MRI techniques are limited to assessing tumour size, infiltration into adjacent anatomical structures and metastatic spread [5]. Diffusion-weighted imaging (DWI) probes into Brownian motion of water molecules in the extracellular space of biological tissues. Thus, DWI provides quantitative data of tissue microstructure, which is thought to represent one aspect of tumour dignity. The clinical impact of DWI for tumour detection and classification has been a subject of on-going research for more than a decade [6]. Available DWI studies on neuroblastic tumours mainly evaluated the feasibility of apparent diffusion coefficient (ADC)-based differentiation of tumour subtypes [7–9], but also demonstrated diagnostic utility for detecting metastases and monitoring therapy response [10–12].

In our study, we focussed on quantitative DWI as a potential non-invasive predictor of tumour dignity and survival. We performed quantitative analyses on 3-T MRI data from a paediatric cohort of patients with neuroblastic tumours in order to investigate the association between imaging characteristics, risk factors and clinical course of disease.

## Methods

### Study design

**Fig. 1:**
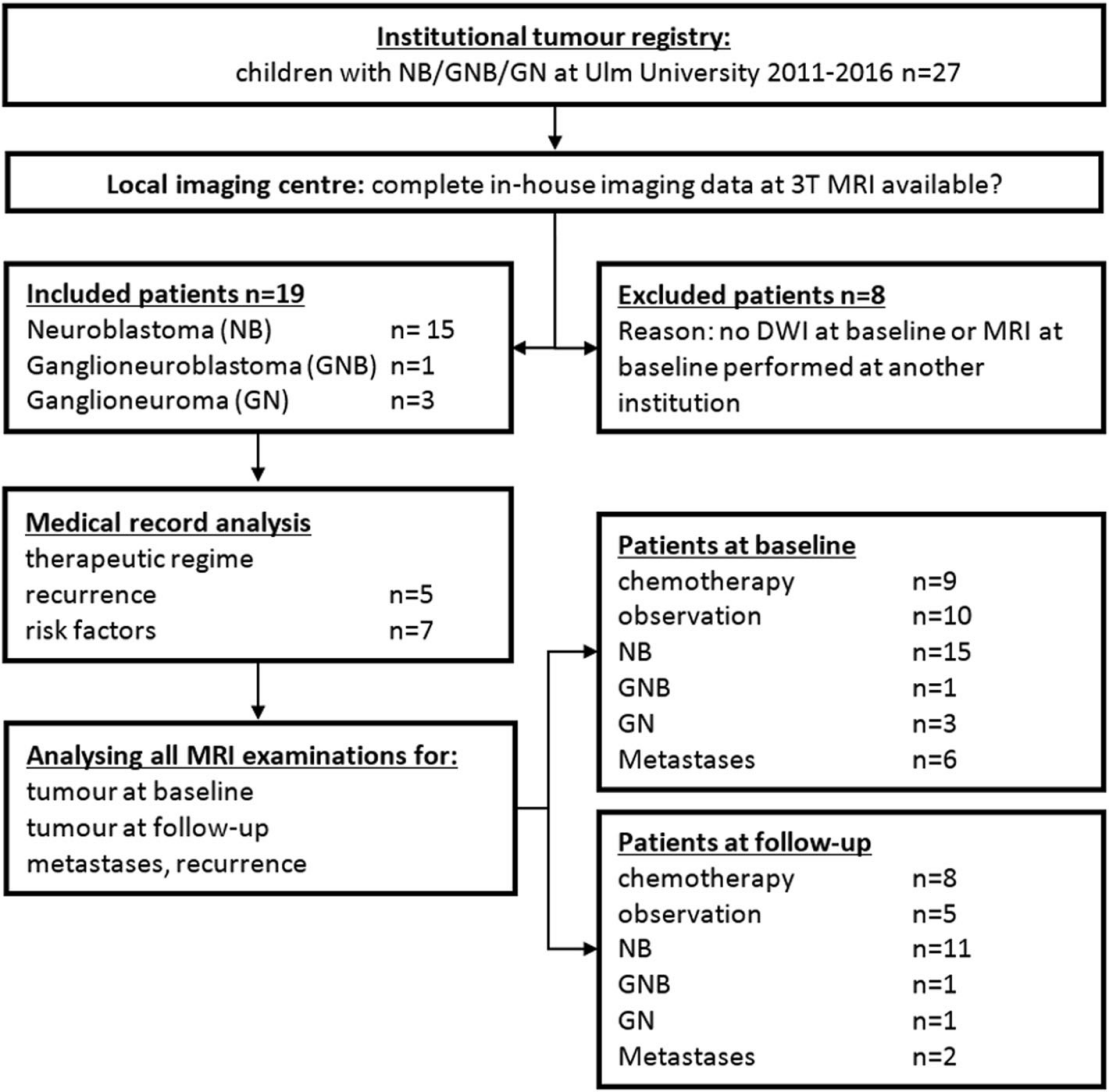
Flowchart highlighting the process of patient selection and the patient subgroups for data analysis

We retrospectively identified a study cohort comprising all paediatric patients with primary diagnosis of a neuroblastic tumour over a period of five years at our tertiary care centre. The study was approved by the institutional review board. Informed consent was obtained from all patients and/or their legal guardians for all diagnostic measures. The following inclusion criteria were used: primary diagnosis of a neuroblastic tumour without prior tumour therapy, histopathological data available, and complete sets of in-house MRI at primary diagnosis, including DWI. All eligible patients were consecutively included in this retrospective single-centre cohort study. Figure 1 illustrates patient enrolment and exclusion.

### Patients

Our study group comprises 19 patients: 15 children with NB, three with GN and one with GNB. Mean age at first diagnosis was 12 ± 41 months (range 1 month–13 years), without gender preference (female = 10; male = 9). Tumour localisation was cervical (*n* = 2), mediastinal (*n* = 3) and abdominal (*n* = 14). Risk factors included bone-marrow metastases (*n* = 4), MYCN-amplification (*n* = 3) and 1p deletion (1p deletion *n* = 4). Information on tumour stage at first diagnosis (low risk, *n* = 12; medium risk, *n* = 2; and high risk, *n* = 5), relapsing disease and therapeutic regime were retrieved from medical history. All children underwent treatment in accordance with the “NB2004 Protocol for Risk Adapted Treatment of Children with Neuroblastoma” of the German Association for Paediatric Oncology and Haematology.

### MRI examination and analysis

All patients were examined on a 3-T scanner (Magnetom Skyra, Siemens Healthineers; Erlangen, Germany) with phased-array body coils. Standard unenhanced and contrast-enhanced sequences (coronal T2-weighted turbo inversion-recovery magnitude, transverse T2-weighted half Fourier acquisition single-shot turbo spin-echo and/or T2-weighted turbo spin-echo, and unenhanced/contrast-enhanced T1-weighted turbo spin-echo sequences with weight-adapted standard dose of gadolinium-based contrast agent) and DWI were acquired using the same standardised examination protocol. DWI was performed prior to intravenous injection of contrast agent with the following parameters: free-breathing transverse fat-saturated single-shot echo-planar imaging; repetition time for neck imaging, 5200 ms; repetition time for thoracic and abdominal imaging, 6000 ms, echo time 71 ms; *b* values 0, 400 and 800 s/mm^2^, slice thickness 5 mm). In patients younger than 6 years of age, that is 17 out of 19 patients in our study, intravenous sedation was administered and monitored by a paediatric anaesthesiologist.

**Fig. 2:**
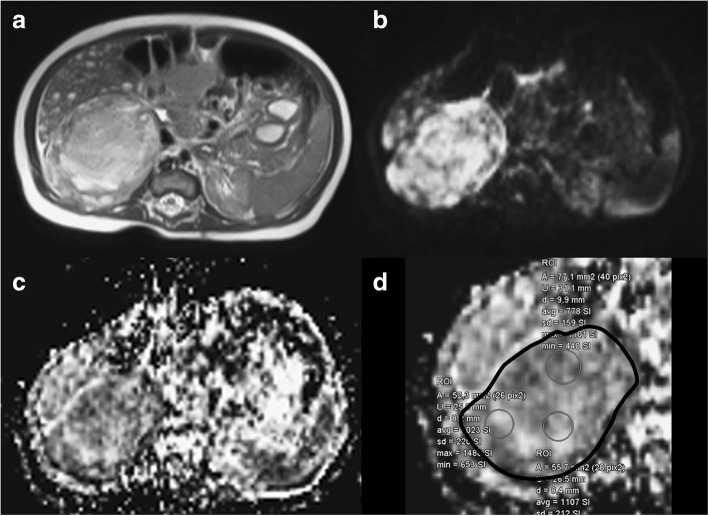
Baseline MRI of a 5-week-old boy with neuroblastoma of the right adrenal gland and suspected multiple liver metastases shown on T2-weighted image (**a**), DWI with *b* = 800 s/mm^2^ (**b**), and ADC map (**c**). To demonstrate the large region of interest (ROI) technique used in our study for ADC quantification, **d** presents a magnification of **c** with a solid black line as overlay marking the free-hand ROI drawn along the tumour margins. Mean ADC of the tumour was measured as 0.883 × 10^−3^ mm^2^/s, and the patient did not suffer a tumour relapse. Three additional small circular ROIs yielded ADC values from 0.778 to 1.107 × 10^−3^ mm^2^/s, indicating some ADC variability in the visually rather homogeneous tumour. The small ROI data were not used in the present analysis

**Fig. 3:**
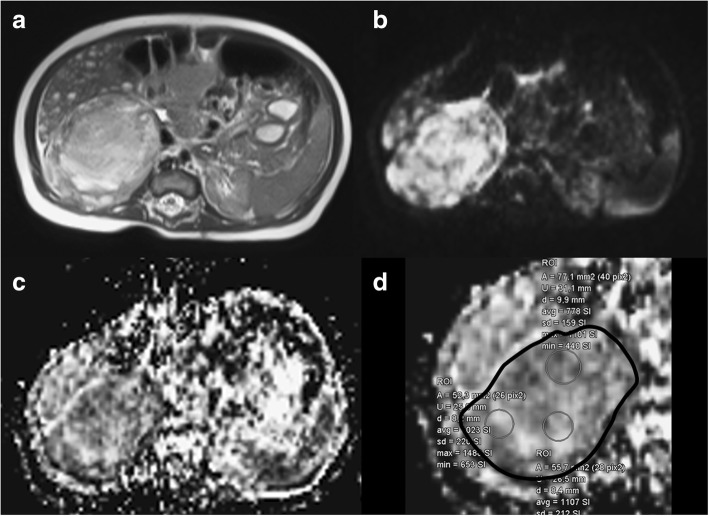
Baseline MRI of a 16-month-old boy with neuroblastoma of the left adrenal gland and suspected liver metastases (liver lesions not shown). The tumour signal is notably more heterogeneous than that of the lesion shown in Fig. 2 and comprises some high-signal areas on the T2-weighted image (**a**). In **b**, a high signal on DWI (*b* = 800 s/mm^2^) can be appreciated while a low signal is visible on the ADC map (**c**) is, however, predominant within the tumour. The presented cross-sectional image was chosen for analysis, as the cross section at the largest tumour diameter included a higher proportion of heterogeneous tumour signal. The black solid line overlay on the magnified ADC map highlights the large ROI drawn for study purposes (**d**). Mean baseline ADC was measured as 0.706 × 10^−3^ mm^2^/s in this eventually relapsing tumour. Three small ROIs yield ADC values of 0.589, 0.638, and 2.841 × 10^−3^ mm^2^/s, the latter equalling liquefied tumour necrosis

Image analysis was performed on a dedicated radiological workstation (Impax EE R20, Agfa Health Care, Germany). Data were collected and analysed by one paediatric radiologist with more than ten years of experience in paediatric DWI. Tumour volume quantification was based on three diameters measured on standard T2-weighted images and was calculated according to the tri-axial ellipsoid formula ($V=\frac{4}{3}\pi abc$). Mean ADC was determined by regions of interest (ROI) measurements using a large ROI approach: a free-hand ROI was drawn at the slice position of the greatest transversal tumour diameter on the ADC map, arranged side-by-side with the corresponding DWI (*b* = 800 s/mm^2^) image on the monitor, to include the total tumour cross-section (Fig. 2). If that cross-section showed significant amounts of necrosis or haemorrhage, we looked for adjacent slices with more homogenous signal and placed the ROI there (Fig. 3). In addition to measurements of the primary tumour, the largest metastasis—if present and quantifiable—was assessed likewise. Mean ADC values were compared between subgroups: NB *versus* GNB/GN, patients with risk factors *versus* patients without risk factors and observation group (low-risk tumours) *versus* therapy group (medium and high-risk tumours).

### Statistical analysis

Data collection and spreadsheet analysis were performed with Microsoft Excel 2010 for Windows. Mean, median, standard deviation and ranges were calculated. Normally distributed data are presented as mean ± standard deviation, and data deviating from normal distribution as median and interquartile range.

All further data analyses were performed with SPSS Statistics 24 for Windows (IBM SPSS Statistics for Windows, Armonk, NY, USA). Non-parametric tests were applied to the small samples in our study. Mann-Whitney *U* test was used to test for significant differences in two independent samples, Wilcoxon test was employed for dependent samples. Receiver operating characteristic (ROC) curves were plotted to identify cutoff values for classifying subgroups of patients. Kaplan-Meier-estimator was used for estimation of overall survival and event-free survival.

Confidence intervals of sensitivity, specificity and other parameters of diagnostic performance were calculated as exact binomial confidence intervals according to the Clopper-Pearson method using the software MedCalc Version 18.11 (MedCalc®, MedCalc Software, Belgium).

Values of *p* lower than 0.05 were considered as indicating statistically significant differences.

## Results

All MRI scans were completed safely and without adverse effects to the patients.

### MRI at baseline

**Fig. 4:**
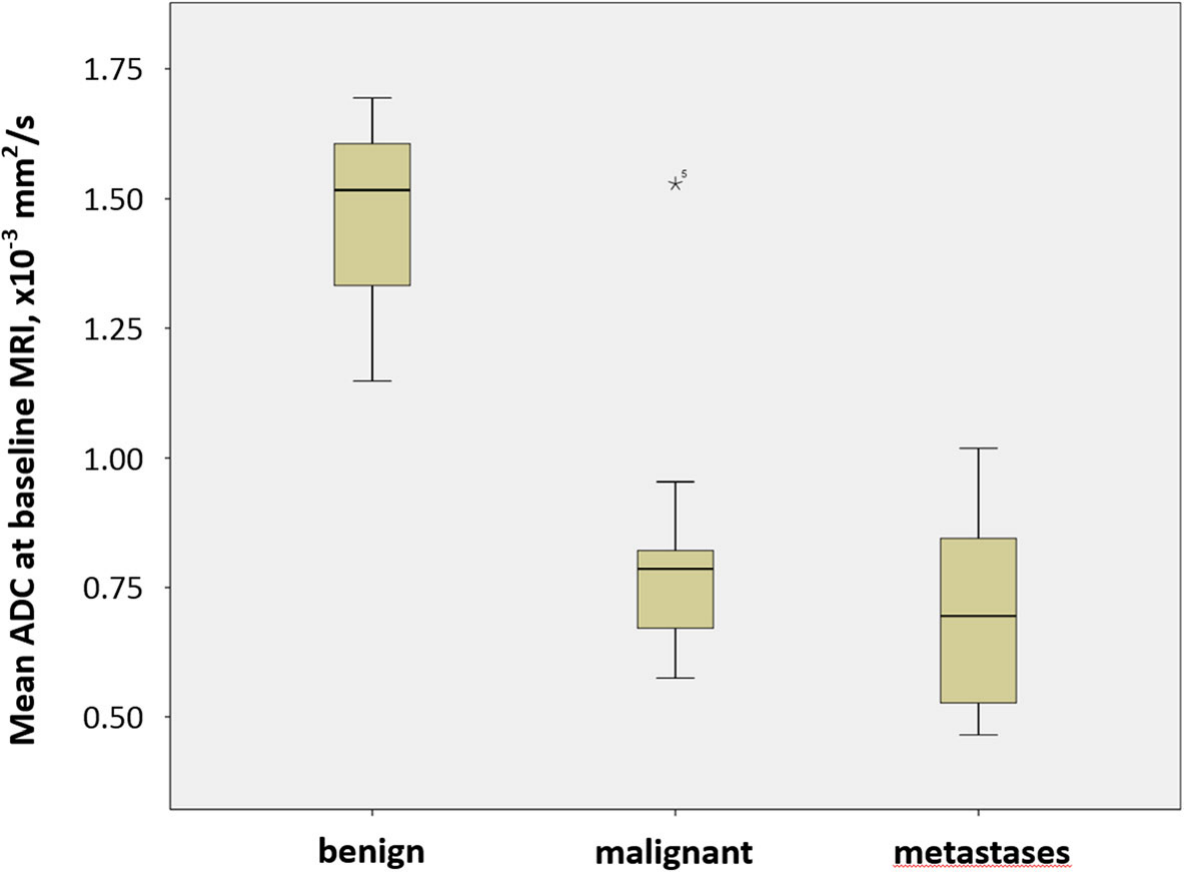
Boxplot of mean tumour ADC of benign lesions (ganglioneuroma, GN, *n* = 3), malignant lesions (neuroblastoma, NB, *n* = 15; ganglioneuroblastoma, GNB, *n* = 1) and untreated metastases (*n* = 6) at baseline. The one outlier in the category “malignant” represents the one case of malignant GNB in our study

At first diagnosis, mean ADC was significantly lower in NB (*n* = 15), compared to GNB/GN (*n* = 4) (0.76 ± 0.11 *versus* 1.47 ± 0.23 × 10^−3^ mm^2^/s; *p* = 0.003) without overlap between the two groups (Table 1, Fig. 4). The one malignant GNB in our patient cohort had a mean ADC value of 1.53, similar to that of the GN. ROC analysis identified a cutoff value for mean ADC of 1.05 × 10^−3^ mm^2^/s to distinguish between malignant (NB and GNB) and non-malignant neuroblastic tumours (GN), with a sensitivity of 100.0% (95% confidence interval 29.2–100.0%) and a specificity of 93.8% (95% CI 69.8–99.8%). The area under the curve was 0.958 with a standard error of 0.047 and 95% confidence interval ranging from 0.867 to 1. Excluding the one case with GNB from analysis, any cutoff ADC value between 0.96 and 1.14 × 10^−3^ mm^2^/s separated malignant from non-malignant lesions without misclassified cases.

**Table 1:** ADC values of tumour entities and subgroups at baseline and during follow-up

	ADC baseline	ADC follow-up
NB all	*n* = 15	*n* = 11
	0.76 ± 0.11	0.89 ± 0.29
	(0.58–0.95)	(0.49–1.49)
GNB + GN	*n* = 4	*n* = 2
	1.47 ± 0.23	GNB 0.90
	(1.15–1.69)	GN 1.84
NB with therapy	*n* = 6	*n* = 5
	0.70 ± 0.08	0.71 ± 0.25
	(0.58–0.79)	(0.49–1.14)
NB with observation	*n* = 7	*n* = 4
	0.82 ± 0.09	0.93 ± 0.12
	(0.65–0.95)	(0.77–1.06)
NB with risk factors	*n* = 6	*n* = 5
	0.70 ± 0.08	0.72 ± 0.25
	(0.58–0.79)	(0.78–1.50)
NB without risk factors	*n* = 9	*n* = 7
	0.79 ± 0.11	1.04 ± 0.23
	(0.59–0.95)	(0.77–1.50)

*ADC* apparent diffusion coefficient, *GN* ganglioneuroma, *GNB* ganglioneuroblastoma, *NB* neuroblastoma

Data is presented as mean ± standard deviation and range (minimum–maximum)

Mean ADC was somewhat lower in NB patients with risk factors (*n* = 6) than in NB patients without risk factors (*n* = 9) without a statistically borderline difference (*p* = 0.077). NB patients who eventually had tumour resection plus observation or observation only showed a significantly higher mean ADC values at baseline when compared to NB patients treated with chemotherapy (*p* = 0.032). Metastases were present in six patients at first diagnosis with a mean ADC of 0.71 ± 0.22 × 10^−3^ mm^2^/s. We observed a borderline difference in ADC values comparing metastases seen in six patients in regional lymph node (*n* = 3), liver (*n* = 2) and bone (*n* = 1) with the corresponding primary tumours (0.71 ± 0.22 × 10^−3^ mm^2^/s *versus* 0.88 ± 0.33 × 10^−3^ mm^2^/s, *p* = 0.078).

### Follow-up MRI

Three months after the initial scan, follow-up MRI showed a significant reduction in median tumour volume in 13 of those 14 patients who had not undergone surgical resection by that time (baseline 354 mL *versus* follow-up 113 mL, *p* = 0.004). Increasing tumour volume was observed in one patient with GN with a tumour volume of 682 mL at first presentation, 777 mL after three months and 791 mL after another round of follow-up at twelve months, without any further signs of tumour progression since that time.

Mean ADC showed a small, but statistically significant increase at follow-up in five GN and NB patients under observation only (baseline 1.0 ± 0.40 × 10^−3^ mm^2^/s *versus* follow-up 1.11 ± 0.42 × 10^−3^ mm^2^/s, *p* = 0.043). There was no progressive disease or relapse observed in this group so far with follow-up ranging from 8 to 56 months.

Tumour volume decreased between baseline and follow-up in all seven patients who had chemotherapy. Among these cases, there were five medium-risk and high-risk patients who subsequently suffered a tumour recurrence in spite of an initial therapy-induced reduction in tumour volume between 48% and 88%. In three patients with tumour relapse during chemotherapy, ADC showed a tendency of further decrease without statistically significant difference in this small sample (baseline 0.95 ± 0.51 × 10^−3^ mm^2^/s *versus* follow-up 0.71 ± 0.2 × 10^−3^ mm^2^/s, *p* = 0.170). Evaluation of the patients with chemotherapy and without relapse during ensuing observation demonstrated a not significant increase in mean tumour ADC (baseline 0.71 ± 0.06 × 10^−3^ mm^2^/s *versus* follow-up 0.80 ± 0.30 × 10^−3^ mm^2^/s, *p* = 0.590).

### ADC and prognosis

**Fig. 5:**
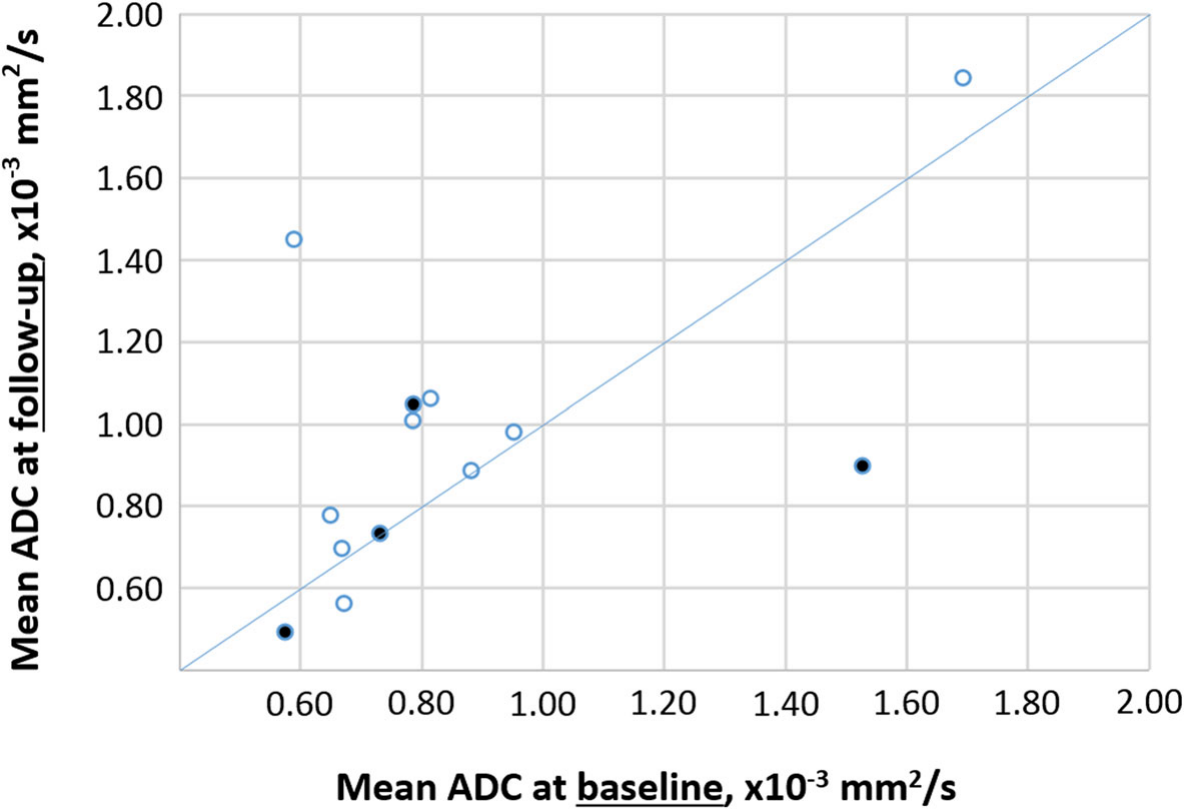
Scatter plot of tumour ADC at baseline and on follow-up for 13 patients, including 11 patients with neuroblastoma (NB), one patient with ganglioneuroblastoma (GNB) and one patient with ganglioneuroma (GN). The filled markers indicate patients who suffered tumour relapse, the markers without filling represent patients without recurrence. Of three patients with falling ADC on follow-up (*below the line*), two had relapsing tumours (66%). In two patients with constant ADC (*on the line*), there was one recurrence (50%). Among eight patients with ADC increase on follow-up (*above the line*), only one tumour relapsed (13%)

**Fig. 6:**
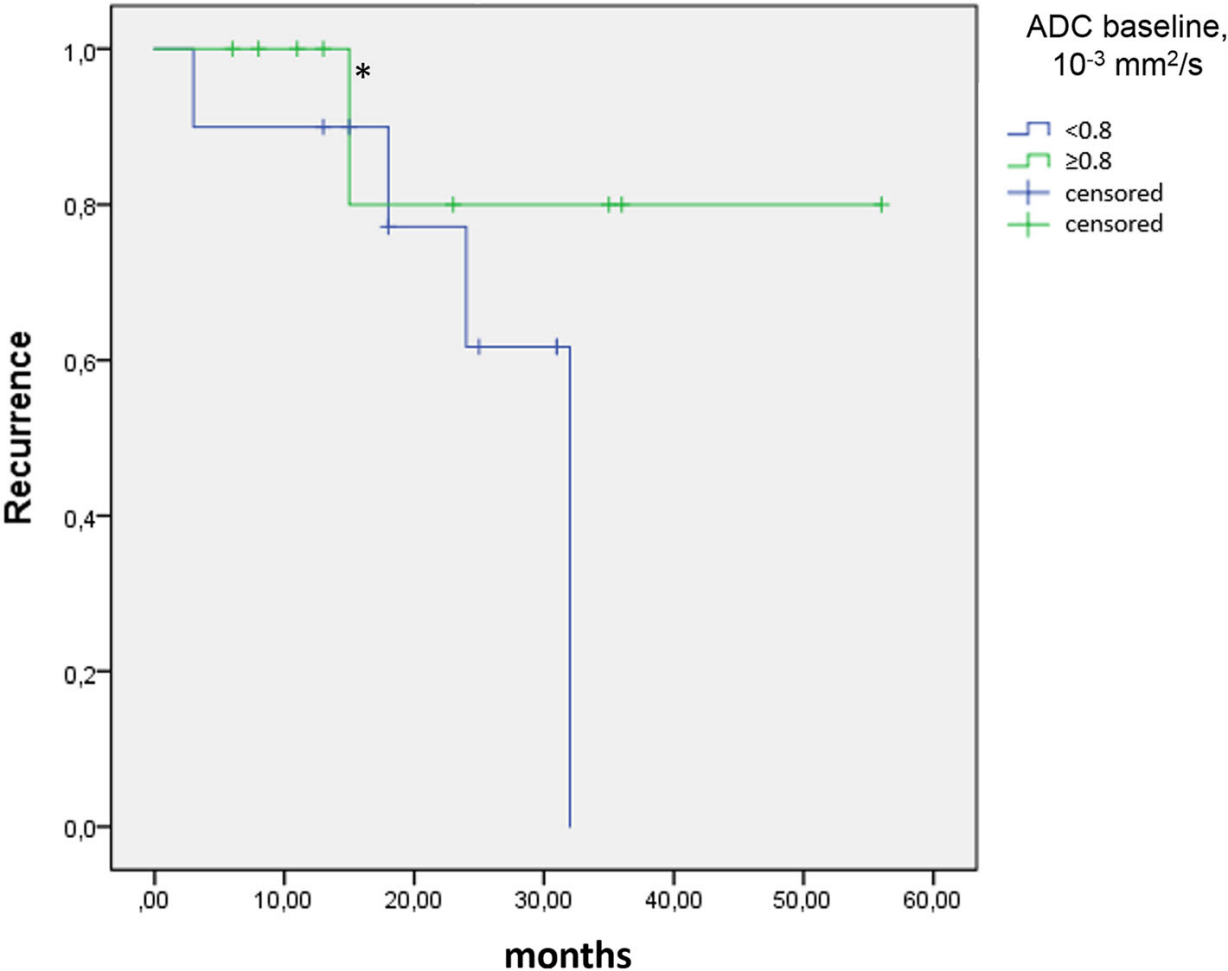
Event-free survival Kaplan-Meier estimate for all patients (*n* = 19) in our study using a cutoff baseline ADC ≥ 0.80 10^−3^ mm^2^/s. The initial sample size showed an ADC ≥ 0.80 mm^2^/s for nine patients and an ADC < 0.80 10^−3^ mm^2^/s in ten patients. The asterisk (*) indicates the recurrence of the malignant ganglioneuroblastoma

**Fig. 7:**
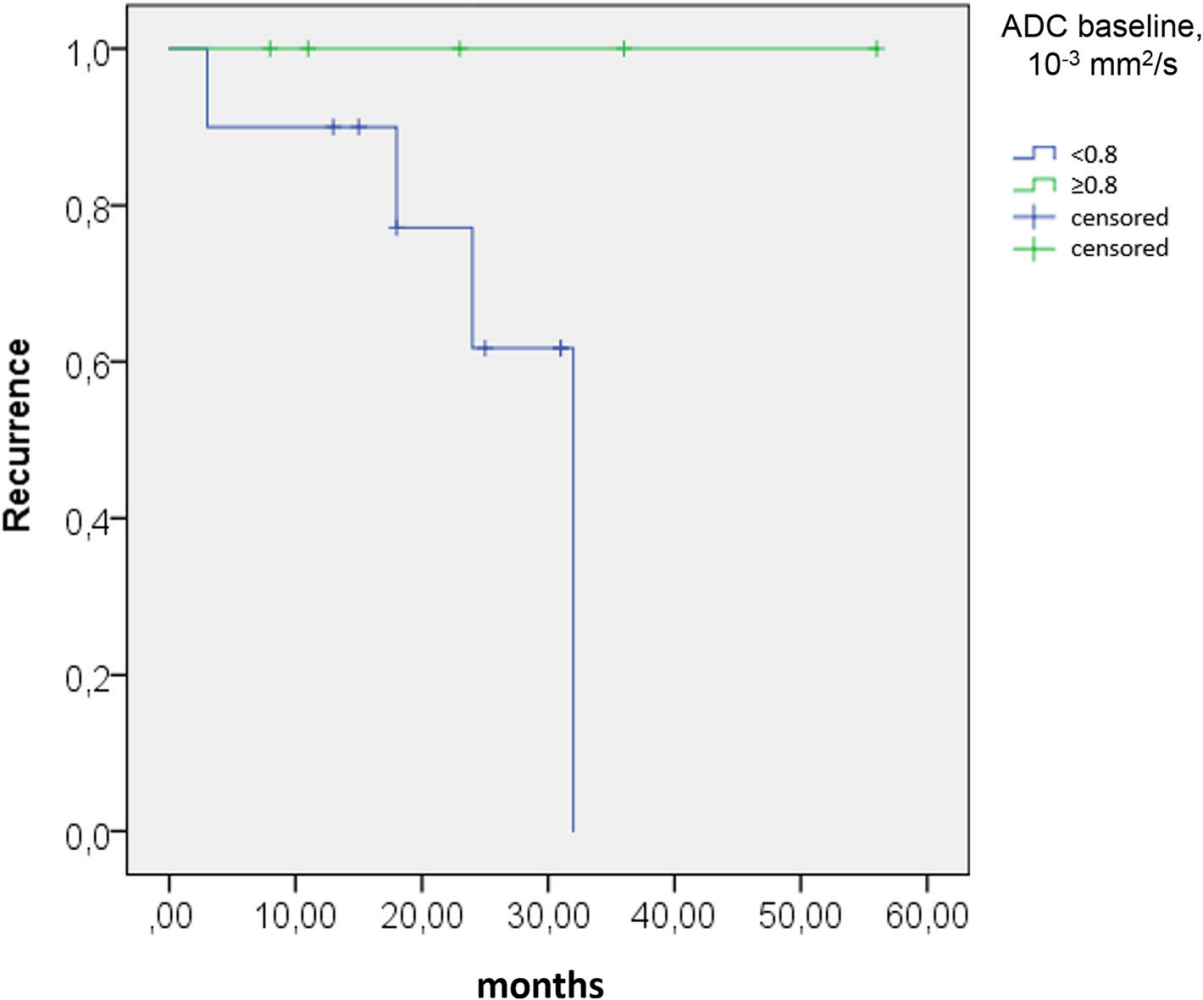
Kaplan-Meier estimate for neuroblastoma patients (*n* = 15) using a cutoff baseline ADC ≥ 0.80 × 10^−3^ mm^2^/s. The initial sample size with an ADC ≥ 0.8 is five patients and with an ADC < 0.80 × 10^−3^ mm^2^/s is ten patients

Patients with constant or falling ADC on follow-up, compared to baseline, experienced tumour recurrence in a higher percentage of cases (Fig. 5). Correlating ADC at baseline with the subsequent occurrence of tumour relapse, all four NB patients with tumour relapse had a mean ADC at baseline < 0.80 × 10^−3^ mm^2^/s, with the exception of the relapsing malignant GNB which had an initial ADC of 1.53 × 10^−3^ mm^2^/s. ROC analysis confirmed a mean ADC value of 0.80 × 10^−3^ mm^2^/s at baseline as the optimal cutoff for distinguishing study patients with and without relapsing tumour. This cutoff value provides an 80.0% specificity (95% confidence interval [CI] 28.4–99.5%) and 57.1% sensitivity (95% CI 28.9–82.3%), with an area under the curve of 0.59. Using the same cutoff ADC on NB patients only, ROC analysis yields 100.0% specificity (95% CI 47.8–100.0%) and 40.0% sensitivity (95% CI 12.2–73.8%) with an area under the curve of 0.65. Based on the cutoff ADC value of 0.80 at baseline, the Kaplan-Meier plots show distinctly different curves for event-free survival in all study patients and in study patients with NB only (Figs. 6 and 7). Of the 19 patients in our study cohort, three patients died at 29 to 45 months after initial presentation, all three patients having a baseline tumour ADC lower than 0.80 × 10^−3^ mm^2^/s.

Comparing baseline tumour ADC < 0.80 × 10^−3^ mm^2^/s and the clinical risk group (intermediate and high risk) as predictors of tumour recurrence, the parameters of diagnostic performance are as follows: specificity 57.1% (95% CI 28.9–82.3%) vs. 78.6% (95% CI 49.2–95.3%), sensitivity 80.0% (95% CI 28.4–99.5%) vs. 80% (95% CI 28.4–99.5%), positive predictive value 40.0% (95% CI 24.0–58.5%) vs. 57.1% (95% CI 30.9–79.9%), negative predictive value 88.9% (95% CI 56.7–98.0%) vs. 91.7% (95% CI 65.1–98.5%) and diagnostic accuracy 63.2% (95% CI 38.4–83.7%) vs. 79.0% (95% CI 54.4–94.0%).

## Discussion

Our study provides first and preliminary evidence that not only does quantitative DWI reliably differentiate between malignant and benign neuroblastic tumours, but it may also provide prognostic biomarkers for evaluating therapy response as well as predicting risk of tumour recurrence.

One of the earliest published clinical applications of extracranial DWI in paediatric patients, as reported by Uhl et al. [13] suggested its diagnostic utility in the work-up of neuroblastic tumours. Neuroblastoma are characterised by high cellularity, which translates into a high-signal and superior tumour-to-background contrast as a proxy of lesion conspicuity on DWI [10]. Just as tissue microstructure varies between different types of neuroblastic tumours with positive correlation of cellularity and malignant potential [14], so does the degree of restricted diffusivity as quantified by the ADC. Consequently, available studies consistently reported significant differences in ADC between NB and GN, with intermediate ADC values for GNB as an entity of mixed histological composition and of variable malignant potential. Gahr et al. [9] and Serin et al. [8] found some overlap between groups of malignant and benign disease in cohorts of 16 and of 24 patients, respectively. In contrast, another study on 29 patients with neuroblastic tumours, including four GBN [10] reported no overlap. A recently published report on 25 children with thoracoabdominal neuroblastic tumours examined with DWI at 3 T further refined analysis and correlated ADC with various histological subtypes and differentiation grades [15]. The overall picture is that of a continuous spectrum of disease, rather than of discrete categories, both on the histopathological level and on diagnostic imaging.

The results of our present study are in line with earlier findings in this respect. The ADC cutoff value of 1.05 identified on ROC analysis for differentiating malignant from benign disease falls exactly within the range proposed in an earlier study that used hardware of the same manufacturer, but analysed DWI scans acquired at 1.5 Tesla with somewhat different scan parameters and different techniques of ROI measurement [10].

A new aspect from our study is the use of ADC as an indicator of therapy response. The most commonly used quantitative parameter for measuring tumour response to therapy in a clinical setting is tumour size, or tumour volume. Yoo et al. [16] state that a therapy-induced decrease in tumour volume by > 40% is a strong predictor of event-free 5-year-survival in patients with high-risk neuroblastoma. In our study, tumour volume significantly decreased in all intermediate/high-risk NB/GNB patients with therapy. However, five of the seven patients in this group eventually suffered tumour recurrence in spite of tumour shrinkage of 48% and higher between baseline and follow-up scan. Interestingly, three of these patients with early relapse were seen with falling ADC values at follow-up, while ADC tended to increase at follow-up in low-risk NB patients under observation. Therefore, changes in tumour ADC measured during, or after, chemotherapy may outperform volume quantification as a non-invasive predictor of therapy response, similar to what has been reported from other malignancies in adult patients [17].

With regard to ADC as a possible predictor of event-free survival, baseline ADC < 0.80 × 10^−3^ mm^2^/s is as sensitive as a biomarker as the clinical risk group classification and carries a similar high negative predictive value, based on our data. We observed no tumour recurrence in our NB patients during the available observation period if baseline ADC were 0.80 × 10^−3^ mm^2^/s or higher. Such association between ADC and disease-free survival has, to our best knowledge, not been reported in paediatric tumours before. However, there is supportive data from adult cohorts, where in a head-to-head comparison of DWI and positron emission tomography/computed tomography (PET/CT), ADC performed as well as maximum standardised uptake value in predicting disease-free survival in patients with head and neck squamous cell carcinoma [18]. In a paediatric diagnostic setting, one would, of course, prefer MRI to PET/CT for risk stratification if both methods performed equally well.

Our study suffers methodological limitations mainly arising from the retrospective study design and from the small total number of patients enrolled. Although counting among the paediatric tumours with the highest incidence, neuroblastoma is nevertheless rare with a population-based age-adjusted incidence rate of 1.3 per 100.000 in Germany [19]. The number of patients treated in any single centre over a couple of years is too small to accumulate into a major cohort. Small sample size translates into a low power of statistical tests. For instance, Kaplan-Meier analysis of a test cohort and a validation cohort, as well as a Cox regression analysis, were not considered feasible in this small sample. Our findings are therefore to be considered very preliminary and are best evaluated in the context of other studies published on other small cohorts. Despite all differences in technical setup and analytic techniques, compared to earlier research, our results by and large support and underline existing knowledge on DWI of NB and GN. As in other studies, GNB constitutes the smallest, and the most volatile, subgroup. While the only GNB patient in our cohort exhibited some imaging features of benign disease, the clinical presentation with synchronous lymph node metastasis and subsequent tumour recurrence was strikingly malignant. The yet unclarified relation between biological potential of GNB and their features on diagnostic imaging and on histopathology invites further investigations.

Quantitative data collected by more than one observer or repeated analysis by the same reader would have allowed for evaluation of interobserver and intraobserver variability, as reported in an earlier publication [10], but were not available in this study. Our readings were performed by one experienced paediatric radiologist with the knowledge that previously published data demonstrated low variability in ADC quantification, even with readers at different levels of experience [10].

In conclusion, based on the new preliminary evidence from our study, we hold that DWI has already become a valuable and reliable imaging tool and may develop into a new risk biomarker for the diagnostic work-up of patients with neuroblastic tumours. Low ADC at first presentation is a highly characteristic of malignancy. Low, and falling, ADC with chemotherapy seems to predict early relapse, and perhaps poor outcome, in spite of therapy-induced reduction in tumour volume. Finally, in the light of ongoing discussions concerning the long-term safety of gadolinium-based contrast agents [20], DWI may help to avoid contrast agent administration, if scanned as a substitute, not as a supplement, to contrast-enhanced sequences, for instance at follow-up. Available data on image quality, lesion conspicuity and diagnostic performance seem to support such considerations [10], although verification of these preliminary findings through further studies would be welcome.

## Abbreviations

ADC: Apparent diffusion coefficient
DWI: Diffusion-weighted imaging
GN: Ganglioneuroma
GNB: Ganglioneuroblastoma
MRI: Magnetic resonance imaging
NB: Neuroblastoma
ROC: Receiver operating characteristic
